# Enzymatic Synthesis and Flash Chromatography Separation of 1,3-Diferuloyl-*sn*-Glycerol and 1-Feruloyl-*sn*-Glycerol †

**DOI:** 10.3390/mps3010008

**Published:** 2020-01-16

**Authors:** David L. Compton, Michael Appell, James A. Kenar, Kervin O. Evans

**Affiliations:** 1 Renewable Product Technology Research Unit, United States Department of Agriculture, Agricultural Research Service, National Center for Agricultural Utilization Research, 1815 N. University Street, Peoria, IL 61604, USA; kervin.evans@usda.gov; 2 Mycotoxin Prevention and Applied Microbiology, United States Department of Agriculture, Agricultural Research Service, National Center for Agricultural Utilization Research, 1815 N. University Street, Peoria, IL 61604, USA; michael.appell@usda.gov; 3 Functional Foods Research Unit, United States Department of Agriculture, Agricultural Research Service, National Center for Agricultural Utilization Research, 1815 N. University St., Peoria, IL 61604, USA; jim.kenar@usda.gov

**Keywords:** diferuloyl glycerol, feruloyl glycerol, flash chromatography, transesterification

## Abstract

Ethyl ferulate was transesterified with Enova Oil (a soy-based vegetable oil containing 80–85% diacylglycerol) using Novozym 435 at 60 °C. The resultant feruloylated vegetable oil reaction product produced a precipitate (96.4 g, 4.02 wt%) after 7 d of standing at room temperature. Preliminary characterization of the precipitate identified the natural phenylpropenoids 1,3-diferuloyl-*sn*-glycerol (F_2_G) and 1-feruloyl-*sn*-glycerol (FG) as the major components. A flash chromatography method was developed and optimized (e.g., mass of sample load, flow rate, binary solvent gradient slope, and separation run length) using a binary gradient of hexane and acetone mobile phase and silica gel stationary phase to separate and isolate F_2_G and FG. The optimized parameters afforded F_2_G (1.188 ± 0.052 g, 39.6 ± 1.7%) and FG (0.313 ± 0.038 g, 10.4 ± 1.3%) from 3.0 g of the transesterification precipitate, *n* = 10 trials. Overall, all flash chromatography separations combined, F_2_G (39.1 g, 40.6%) and FG (9.4 g, 9.8%) were isolated in a combined yield of 48.5 g (51.4%), relative to the 96.4 g of transesterification precipitate collected. The optimized flash chromatography method was a necessary improvement over previously reported preparative HPLC and column chromatography methods used to purify milligram to low gram quantities of F_2_G and FG to be able to process ~100 g of material in a timely, efficient manner.

## 1. Introduction

The phenylpropenoid, ferulic acid, is a 4-hydroxy-3-methoxy-substituted cinnamic acid and is a nearly ubiquitous component throughout the higher plant kingdom, and as such is a common dietary component [1]. In plants, it can play various roles, but mostly it is expressed as a structural component of hemicellulose and lignin in the plant cell wall and in suberin and waxy surfaces of leaves and other plant parts. Ferulic acid has also been found as a natural constituent in plants esterified to phytosterols in grain brans [2], to sugars in herbs and plant stalks [3,4], and to glycerol in tubers and flowers [5,6,7]. The highly conjugated structure of ferulic acid (and its esters) imparts it with a strong ultraviolet (UV) absorbing character [8,9]. The presence of a phenolic hydroxyl group also imparts an antioxidant capability to the molecule [10]. These functional traits have long been recognized as being valuable attributes to be exploited for food, health, and personal care applications.

**Figure 1:**
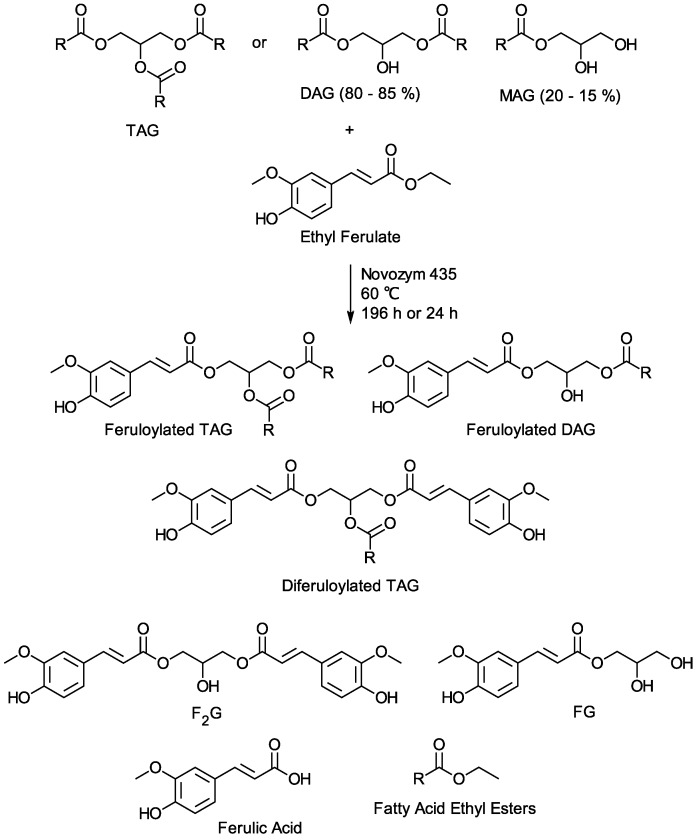
Schematic of the transesterification of vegetable oil TAG or vegetable oil mixture of DAG and MAG with ethyl ferulate, R = vegetable oil fatty acid (e.g., 18:0, 18:1, 18:2, 16:0). Major products: feruloylated triacylglycerol (TAG), feruloylated diacylglycerol (DAG), and diferuloylated TAG. Minor products: feruloylated glycerol (FG), diferuloylated glycerol (F_2_G), and ferulic acid. The product mixture also contains unreacted ethyl ferulate, unreacted vegetable oil, and vegetable oil fatty acid ethyl esters.

Mono- and diferuloyl glycerol, FG and F_2_G (Figure 1), are naturally occurring phenylpropenoids that have been shown to provide UV photoprotection and antioxidative protection to pollen, spores, and waxy leaves in plants [9,11]. FG and F_2_G have also been shown to provide antioxidant protection against free radicals [5,10] and partition into phospholipid vesicles, providing antioxidant protection to lipids against peroxide radicals [12]. Other phenylpropenoid glycerol and glycoside derivatives have demonstrated biological activities, such as oesterogenic, antitumor, and antiproliferative activities and have been shown to prevent the hydrolysis of polysaccharides [5]. Of agricultural interest, feruloylated saccharides in BMR sorghum stalks have been shown to exhibit insecticidal activity against corn earworm and armyworm, agricultural pests that cause major damage to corn, significantly reducing yield and facilitating the colonization by fungal ear molds that produce mycotoxins harmful to people and animals [13]. To determine whether feruloylated glycerols have similar insecticidal activity, to determine feruloylated glycerols physicochemical properties (e.g., UV absorbance, photostability), and to investigate other potential bioactivities (e.g., antifungal, antitumor) of feruloylated glycerols, a route to larger quantities of purified FG and F_2_G is needed.

Limited quantities of FG and F_2_G have been isolated by extraction from natural plant sources (e.g., wheat, potato, lily) [5,6,7]. The de novo synthesis of feruloyl glycerol has been attempted by the esterification of ferulic acid with glycerol via the Mitsunobu protocol [14]. The esterification of ferulic acid with glycerol through *p*-toluene sulfonic acid catalyzed condensation in refluxing toluene has been demonstrated with limited success [15,16]. The glycerolysis of ethyl ferulate, the ethyl ester of ferulic acid, has been accomplished without solvent and using a variety of functionalized ionic liquids as the catalysts [17,18,19,20]. The enzymatic catalyzed esterification of ferulic acid with glycerol has also been demonstrated [20,21]. While these studies demonstrate that FG and F_2_G can be obtained through extraction of plant components or the direct esterification of ferulic acid (or ethyl ferulate) and glycerol, the methods are limited due to low yields, the need for solvents (e.g., DMSO, toluene, THF), chemical catalysts (e.g., triphenylphosphine, sulfonic acids) or 10- to 100-fold excess of glycerol.

We have developed a pilot-scale (1 metric ton/year) continuously fed, packed bed, enzymatic bioreactor employing a commercial lipase to transesterify ethyl ferulate and vegetable oil triacylglycerols (TAG) (e.g., soybean, coconut) to form a mixture of feruloylated di- and triacylglycerols where there is at least one feruloyl moiety and one fatty acid moiety on the glycerol backbone [22,23]. The process uses soybean oil as the solvent and the feruloylated di- and triacylglycerols are used as obtained from the bioreactor and do not require post-production purification or processing. FG and F_2_G are known byproducts (<1%) of the bioreactor transesterification process but have been considered undesirable due to the lack of a fatty acid moiety on the glycerol. Increased water content in the bioreactor has been shown to increase the amount of hydrolysis biproducts, FG, F_2_G, and ferulic acid [24,25]. In addition, conducting the bioreactor transesterification of ethyl ferulate with a mixture of vegetable oil mono- and diacylglycerols (MAG and DAG, respectively) instead of vegetable oil TAG resulted in higher quantities (~4%) FG and F_2_G. Herein, we report methods for harvesting FG and F_2_G and purifying them in >10 g quantities by flash chromatography, an improvement over the milligram to low gram quantities obtained by preparative HPLC and column chromatography methods reported to date [5,14,26].

## 2. Experimental Design

### 2.1. Materials

Ethyl ferulate (4-hydroxy-3-methoxycinnamic acid ethyl ester) was purchased from Capot Chemical Co., Ltd. (Hangzhou, Zhejiang, China). Enova Oil (a vegetable oil containing 80–85% diacylglycerol (DAG) from soybean oil) [27] was purchased from ADM KAO, LLC. (Decatur, IL, USA). Novozym 435 (*Candida antarctica* lipase B immobilized on acrylic beads) was purchased from Brenntag Great Lakes, LLC. (Wauwatosa, WI, USA). All solvents were HPLC grade and purchased from Sigma-Aldrich (St. Louis, MO, USA). RediSep RF Gold Silica Gel Disposable Flash Chromatography Columns (20–40 microns), 4 g, 12 g, 24 g, 120 g, 220 g, and 330 g, were purchased from Teledyne-Isco (Lincoln, NE, USA). See Table 1 for manufacturer’s specifications and recommended parameters.

### 2.2. Equipment

- Model OPF-10 cooking oil filter (Chard, Two Rivers, WI, USA);
- Whatman54 filter paper (Fisher Scientific, Pittsburgh, PA, USA);
- CombiFlash Rf 200i flash chromatography system with UV detection (Teledyne ISCO, Inc. Lincoln, NE, USA);
- Shimadzu HPLC system, consisting of a LC-30AD pump, SIL-20AXR Prominence autosampler, CBM-20A communications bus module, and SPD-M20A Prominence photodiode array detector (Kyoto, Japan);
- Eppendorf CH-500 column heater (Hauppauge, NY, USA);
- Phenomenex Luna Phenyl–Hexyl column (5 µm, 250 × 4.6 mm) (Torrance, CA, USA);
- Bruker AVANCE 500 NMR spectrometer (500 MHz ^1^H/125.77 MHz ^13^C) using a 5 mm broadband inverse (BBI) probe (Billerica, MA, USA);
- Shimadzu UV-1280 UV–Vis spectrophotometer (Shimadzu Scientific Instruments, Inc., Addison, IL, USA);
- Rainin pipettes (Sigma-Aldrich, St. Louis, MO, USA);
- Q2000 Differential Scanning Calorimetry (TA instruments, New Castle, DE).

## 3. Procedure

### 3.1. Synthesis of Feruloylated Soy Glycerides

The pilot-scale, Novozym 435-catalyzed transesterification of ethyl ferulate with vegetable oils (e.g., soybean oil, coconut oil) using a continuously fed, packed bed, enzymatic bioreactor has been previously described [22,23]. A similar, smaller, previously described bioreactor charged with Novozym 435 [24] was employed to transesterify Enova Oil with ethyl ferulate. Batches of Enova oil (2.09 kg) and ethyl ferulate (0.42 kg) were heated to 60–65 °C and filtered through a cooking oil filter (Chard, Two Rivers, WI, USA, Model OPF-10) before being dried for 3–4 h at 60 °C under partial vacuum with a nitrogen sparge and then transferred to the bioreactor feed tank as previously described [22]. The dried, Enova Oil-ethyl ferulate substrate was fed at 1.2 kg/day into the bioreactor through a packed bed column containing 0.4 kg of Novozym 435. The feruloylated vegetable oil product was harvested from the bioreactor and allowed to stand at room temperature for 7 d after which an off white to pale yellow precipitate formed.

The feruloylated Evova Oil product (2.4 kg) containing the precipitate was vacuum filtered through a double layer of Whatman54 filter paper (Fisher Scientific, Pittsburgh, PA, USA) using a Büchner funnel (135 × 60 mm) and a 2 L collection flask. The filter cake was left in the Büchner funnel under vacuum for 18 h, and the crude, yellow precipitate was stored at room temperature. Yield: 96.4 g (4.02 wt% relative to the feruloylated Enova Oil product).

### 3.2. Flash Chromatography Purification of 1-Feruloyl-sn-Glycerol (FG) and 1,3-Diferuloyl-sn-Glycerol (F_2_G)

The crude, yellow precipitate was purified using a Teledyne ISCO, Inc. (Lincoln, NE, USA) CombiFlash Rf 200i flash chromatography system with UV detection at 325 nm, slope-based peak width detection at default for the RediSep column used for separation (Table 1), peak threshold 0.20 absorbance units, and peak collections into 15 mL test tubes. This study varied the RediSep column size, solvent flow rate, solvent gradient, and crude product sample load to optimize the purification of FG and F_2_G. A typical separation consisted of dissolving the crude, yellow precipitate (3.0 g) in acetone (10 mL) and syringe loading the solution onto a 12 g RediSep column. The 12 g RediSep column was aspirated to dryness for 1 h and used as the Load Column plumbed above a 120 g RediSep Separation Column (Figure S1). The separation was conducted at 57 mL/min in “Liquid Load” mode starting with a three-column volume (CV) equilibration of the stacked columns with hexane. The stacked columns were then developed with a 0–80% acetone in hexane gradient over 7 CV, followed by 1 CV of 80% acetone in hexane, and finally 2 CV of 100% hexane. Solvent collection tubes containing target peaks were combined and the solvent removed by rotary evaporation. The resultant white solid (F_2_G) or yellow, oily liquid (FG) were dried under vacuum (11.6 Pa) at 30 °C for 18 h. All separations combined, yield: F_2_G 39.1 g (40.6 wt% relative to the crude, yellow precipitate) and FG 9.4 g (9.8 wt% relative to the crude, yellow precipitate). F_2_G and FG were characterized by HPLC, ^1^H NMR (Figure S2), ^13^C NMR, and LC–ESI–MS, confirming previously published results [5,16,28].

### 3.3. HPLC

Analyses were performed on a Shimadzu (Kyoto, Japan) system, consisting of a LC-30AD pump, SIL-20AXR Prominence autosampler, CBM-20A communications bus module, SPD-M20A Prominence photodiode array detector, Eppendorf (Hauppauge, NY, USA) CH-500 column heater and a Phenomenex (Torrance, CA, USA) Luna Phenyl–Hexyl column (5 µm, 250 × 4.6 mm) kept at 30 °C. Solvents were prepared and filtered using a Whatman 0.45 µm nylon filter (Sigma-Aldrich); Solvent A: water (268 mL), methanol (70 mL), 1-butanol (11 mL) and glacial acetic acid (1 mL); Solvent B: water (93 mL), methanol (245 mL), 1-butanol (11 mL) and glacial acetic acid (1 mL); Solvent C: methanol. Injections (10 µL) were developed on the column at 1.0 mL/min with a 5 min isocratic flow of 3:1 A:B, followed by a 2 min linear gradient to 100% B, followed by a 5 min isocratic flow of 100% B, followed by a 2 min linear gradient to 100% C, followed by a 13 min isocratic flow of 100% C, finally followed by a 3 min linear gradient to 3:1 A:B.

### 3.4. LC–ESI–Mass Spectroscopy

Analyses were conducted on the feruloylated lipid and glycerol samples using previously described methods [23].

### 3.5. NMR Spectroscopy

^1^H and ^13^C NMR spectra were obtained on a Bruker AVANCE 500 spectrometer (500 MHz ^1^H/125.77 MHz ^13^C) using a 5 mm BBI probe. All samples were dissolved in d6-acetone and all spectra were acquired at 27 °C. Chemical shifts are reported as ppm from tetramethylsilane calculated from the lock signal (ΞD = 15.350609%).

### 3.6. UV Spectroscopy

Spectra were recorded on a Shimadzu (Shimadzu Scientific Instruments, Inc., Addison, IL, USA) UV-1280 UV–Vis spectrophotometer in a 1 cm path length, with 3.5 mL volume quartz cuvettes described above, with a scan range of 400–290 nm, scan speed of medium, and scan pitch of 1.0 nm. Stock solutions of F_2_G and FG (1.0 mM) were prepared in ethanol and acetonitrile in 50 mL volumetric flasks and used fresh daily. Serial dilutions of F_2_G (10–50 µL) and FG (10–100 µL) were prepared from each stock solution using Rainin pipettes (Sigma-Aldrich, St. Louis, MO, USA) and 5 mL volumetric flasks. Serial dilutions and UV measurements were conducted in triplicate.

### 3.7. Differential Scanning Calorimetry

Thermal properties were measured using a TA instruments (New Castle, DE, USA) Q2000 DSC calibrated against an indium standard (156.68 °C, 28.86 J g^−1^), equipped with a refrigerated cooling system (RCS 90), and a nitrogen purge gas (50 mL min^−1^). Data sampling and temperature controls were controlled by the “TA Instrument Control” software program. Approximately 6.0–9.0 (±0.1) mg of the samples were weighed into T*_zero_* aluminum DSC pans and hermetically sealed using the corresponding lids. The sealed DSC samples were referenced against an identical empty aluminum pan and run at a linear heating rate (β) of 5.0 °C/min from 0.0 to 160.0 °C. The samples were subsequently cooled to 0 °C at 5.0 °C/min, held 20 min at 0 °C and then reheated across the melting region to obtain the rescanned melting profile. TA Universal Analysis 2000 software, version V4.5 was used to determine the onset temperature (T*_onset_*, °C), peak maximum temperature (T*_peak_*, °C), and enthalpies (ΔH, J g^−1^).

## 4. Results and Discussion

### 4.1. Synthesis of F_2_G and FG

Ethyl ferulate and vegetable oil (e.g., soybean oil, coconut oil) form a miscible solution at 60 °C that can be converted by lipase-catalyzed transesterification to a mixture of feruloylated vegetable oil TAG and DAG [22,23]. The feruloylated vegetable oil TAG and DAG consist of TAG and DAG molecular species with at least one feruloyl moiety exchanged for a fatty acid moiety, covalently attached to the glycerol backbone (Figure 1), which have been identified and quantified by HPLC–MS [26]. The feruloylated glycerol species, F_2_G and FG, having no fatty acid moieties covalently attached to the glycerol backbone and ferulic acid, a hydrolysis product of ethyl ferulate, combined account for <1% of the feruloylated species (Figure 1). Additionally, the final transesterification product mixture contains unreacted ethyl ferulate, the transesterification byproduct, vegetable oil fatty acid ethyl esters, and unreacted vegetable oil TAG. It is also presumed that small quantities of vegetable oil DAG and MAG may be formed from the hydrolysis of the vegetable oil TAG.

The time required to reach ethyl ferulate conversion equilibrium to feruloylated TAG and DAG via lipase-catalyzed transesterification is >140 h [28]. It has been shown that if the lipase-catalyzed transesterification of ethyl ferulate and vegetable oil TAG is conducted in the presence of glycerol [26] or instead using vegetable oil DAG and MAG [24] (Figure 1), the conversion of ethyl ferulate to feruloylated TAG and DAG can reach equilibrium in 24 h; however, the ratio of the feruloylated molecular species is greatly affected. These modified transesterifications result in the increased formation of F_2_G and FG, relative to the other feruloylated molecular species [26]. Herein, the previously commercially available soybean oil-based Enova Oil (≥80% DAG) was transesterified with ethyl ferulate using previously described bioreactor methods [22,24]. After 48 h, the feruloylated Enova Oil mixture harvested from the bioreactor and the combined content of the F_2_G and FG was determined by HPLC to be ~5% relative to the other feruloylated molecular species formed. As anticipated from previous reports [24], after 7 d of standing at room temperature, a precipitate formed in the feruloylated Enova Oil product mixture, which was collected by filtration and the crude precipitate (4 wt% relative to the feruloylated Enova Oil product) was identified by HPLC as a mixture of predominantly F_2_G and FG. FG was not previously identified as a component of the precipitate formed from similar ethyl ferulate transesterifications with vegetable oil DAG and MAG, and the mass yield of the crude precipitate was not reported [24].

### 4.2. Flash Chromatography Separation and Isolation of F_2_G and FG

Flash chromatography, also referred to as low or medium pressure liquid chromatography (typically 25–200 psi, 172–1380 kPa), is primarily a qualitative separation technique used for purification of compounds as opposed to an analytical technique such as HPLC or Ultrahigh HPLC (typically >1000 psi, >6895 kPa) used for quantitative separation and identification of compounds. F_2_G and FG were purified from the raw precipitate obtained from the lipase-catalyzed transesterification of Enova Oil and ethyl ferulate by flash chromatography using silica gel as the stationary phase and a binary gradient of hexane and acetone as the mobile phase. Table 2 summarizes the flash chromatography purification runs attempted using varying raw precipitate (sample) loads, Load Column sizes, Separation Column sizes, flow rates, and resultant F_2_G and FG yields. Before the experimental results detailed in Table 2 are considered, the method used for sample preparation and loading must be discussed. Attempts were made to use the manufacturer’s Solid Sample Cartridge for sample loading, requiring immobilization of the sample onto free silica gel. This was accomplished by rotary evaporating to dryness a silica gel suspension in an acetone solution of the sample (i.e., wet impregnation). The dried, sample-impregnated silica gel was then loaded into the Solid Sample Cartridge and secured with a frit and slide plunger, and the separation runs conducted using the instrument’s ‘Solid Sample’ load mode. This sample loading method was proved unreliable as the frit often slipped out of place under solvent pressure and the slide plunger rubber O-ring sometimes did not seal, both faults causing leaks. A second loading method attempted was using the instruments ‘Liquid Injection’ mode where an acetone solution of the sample was injected directly onto the Separation Column using a disposable syringe and the separation run conducted without a Load Sample Cartridge. This loading method resulted in inconsistent separations as the acetone used to dissolve the sample for loading caused a micro-gradient during the hexane equilibration of the column, causing the sample components (i.e., F_2_G and FG) to move prematurely along the Separation Column before the run’s gradient was applied. The sample loading method used for the flash chromatography separation runs detailed in Table 2 was a modified solid cartridge loading used in conjunction with the instrument’s ‘Liquid Injection” mode, detailed below.

Initial separation runs were conducted on small (100 mg) samples loaded onto a 4 g RediSep column and separated using 24 g RediSep column (Table 2, Run 1 and 2). The sample was dissolved in a minimal amount of acetone and the sample solution injected with a disposable syringe onto the 4 g Load Column. The Load Column was aspirated to dryness under vacuum, and precaution was taken to not use more acetone than could be dried from the Load Column without the solution being drawn completely through and out the bottom of the column. If more acetone was needed to dissolve the sample than the column could accommodate a larger Load Column was used. The sample loaded 4 g RediSep Load Column was plumbed above the 24 g RediSep Separation Column (Figure S1). This modified solid sample loading method was more reliable than the manufacturer’s suggested loading methods, resulting in no plumbing issues, no leaks and consistent separations. The drawback of the modified method, while more robust, is that it required the use of a RediSep column for sample loading which is more expensive than the reusable Solid Cartridge Loader, disposable frits, and relatively cheap silica gel; however, the cost of the RediSep column was defrayed over multiple uses (discussed below).

**Figure 2:**
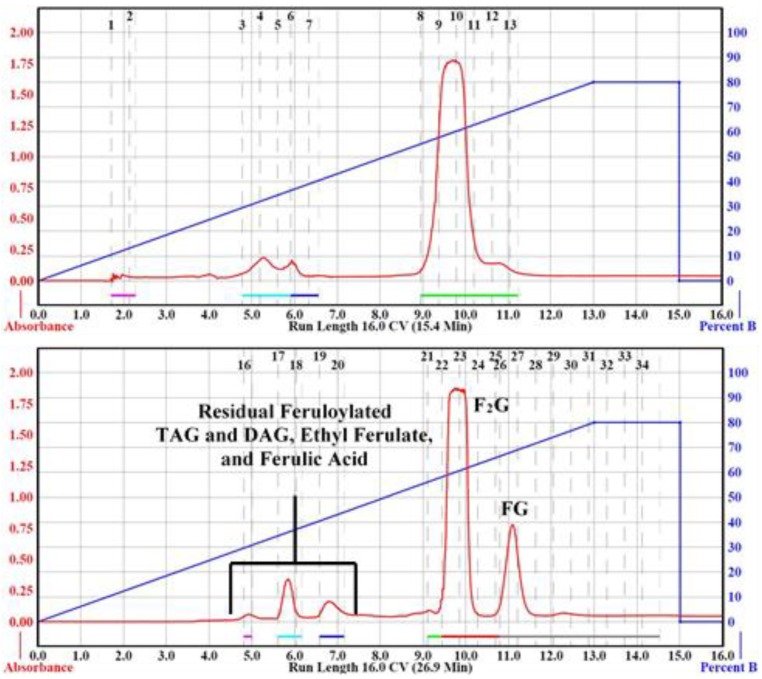
CombiFlash Rf200i flash chromatography Run 1 (**Top**) and Run 2 (**Bottom**) from Table 2. The solvent gradient of Percent B (acetone, right-blue axis) in hexane was denoted as the blue trace and right-blue axis and the signal was monitored by ultraviolet (UV) detection (325 nm, left-red axis, red trace). Black numbers at the top of the chromatograms denote collection tube numbers.

Table 2, Run 1 details the parameters and conditions of the purification of 100 mg sample using the manufacturer’s suggested column specifications (Table 1). The Sample and Load Columns were conditioned with 3 CV of hexane before the binary gradient was applied. The flash chromatography results of Run 1 are depicted in Figure 2. Separation of F_2_G and FG was not obtained using the specified flow rate of 35 mL/min. Several attempts were made to adjust the binary gradient slope and run length using the manufacturer’s suggested flow rate for the 24 g column without success. When the flow rate was arbitrarily reduced by ~1/3 to 20 mL/min using identical parameters separation of F_2_G and FG was obtained. The contents of the collected aliquots were confirmed by HPLC vs. standards (e.g., feruloylated soybean oil, ethyl ferulate, ferulic acid) and F_2_G (calculated exact mass 444.1 u) and FG (calculated exact mass 268.1 u) were further confirmed by LC–ESI–MS as the sodium adduct [M − H + Na], major ion 465.0 *m*/*z*, and the major ion 266.9 [M − H]^−^, respectively, and by ^1^H NMR (Figure S2), confirming previously published results [5,14,20,26]. Table 2, Run 2 was repeated three times and afforded a F_2_G yield of 38 ± 0.025% and a FG yield of 9.4 ± 0.003% (47.4 ± 2.4% total), relative to the initial 100 mg of raw precipitate. This was almost exactly a 4:1 F_2_G:FG ratio isolated from the raw precipitate.

It should be noted that adjusting the flow rate from the manufacturer’s specified flow rate for a RediSep column (Table 1) affected the retention time of a single molecular species as observed by the instrument’s UV detector vs. the instrument’s Evaporative Light Scattering Detector (ELSD). Ethyl ferulate standard (10 mg) was run under identical conditions using a 4 g RediSep Separation Column at four different flow rates (Figure S3). The ELSD signal of the ethyl ferulate corresponds with the UV absorbance signal at 18 mL/min flow rate. However, at lower flow rates, the ELSD signal precedes the UV absorbance detector signal while at higher flow rates the ethyl ferulate ELSD signal succeeds the UV signal. The retention time of the ethyl ferulate UV signal did not appreciably change with changing flow rates; however, the base width of both the ELSD and UV signals increased with increased flow rate. The instrument’s designed flow of the sample after the separation column splits the flow to the two detectors, and precise lengths of tubing are used after the splitter to allow for concurrent ELSD and UV signal detection; however, this timing mechanism for parallel, concurrent detection (tube length) is calibrated to a specific flow rate for each size column. Table S1 shows that the ELSD and UV signals can be offset by as much as 1 CV and peak base widths increased >2-fold by changing the flow rate for a column. Although the study herein required only the use of the UV detector, peak collection of multiple molecular species during a single separation run dependent on both ELSD (for compounds not containing a UV chromophore) and UV peak detection can be affected by changing the flow rate from that specified by the manufacturer for that column.

**Figure 3:**
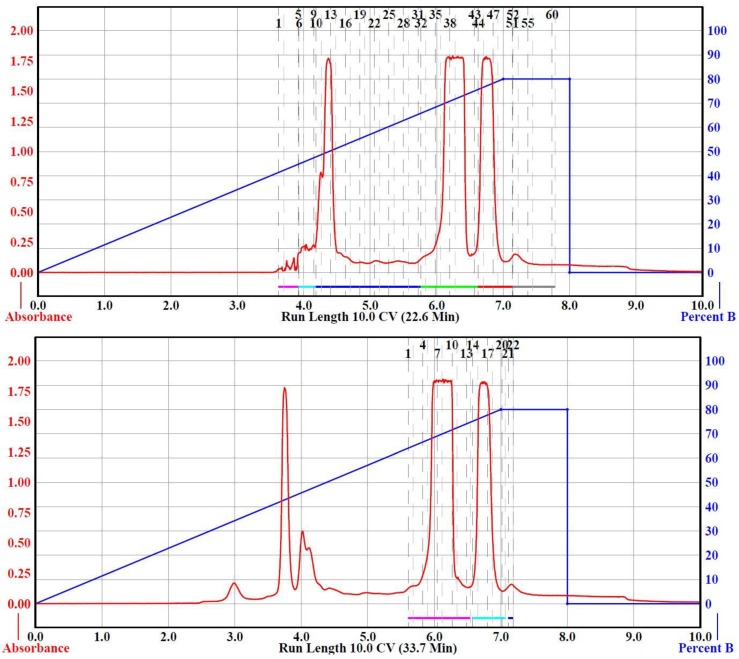
CombiFlash Rf200i flash chromatography Run 3 (**Top**) and Run 4 (**Bottom**) from Table 2. The solvent gradient of Percent B (acetone, right-blue axis) in hexane was denoted as the blue trace and the signal was monitored by UV detection (325 nm, left-red axis, red trace). Black numbers at the top of the chromatograms denote collection tube numbers.

Having shown that small samples (100 mg) of raw precipitate could be separated to isolate F_2_G and FG using Table 2, Run 2 parameters, we experimented to determine the maximum sample load that could be used for the 120, and 330 g RediSep columns to obtain successful separations of F_2_G and FG. Separation of 1, 3, 5, 7, and 10 g loads were attempted with a 120 g RediSep Separation Column (Table 2, Run 3–9), all within the recommended sample loading range (Table 1), using a binary solvent gradient of 0–80% acetone in hexane. The 7 and 10 g loadings required more solvent than the 12 g RediSep Load Column could accommodate; therefore, a 24 g Load Column was used (Table 2, Run 8, 9). Table 2, Run 3 and 4 compared identical parameters for the separation of 1.0 g of sample with Run 3 using the recommended 120 g column flow rate of 85 mL/min and Run 4 using 57 mL/min. As shown in Figure 3, and unlike for the 24 g column (Table 2, Run 1), separation of F_2_G and FG was obtained using the 120 g at the higher, recommended flow rate; however, better base line separation of the two peaks were obtained at the slower flow rate. The yields of the isolated F_2_G and FG were very similar to those obtained from the 100 mg load runs (Table 2). Based on these results, the slower flow rate was used for the higher sample loads for the 120 g Separation Column to ensure F_2_G and FG base line separation. A 3.0 g sample load (Table 2, Run 5) was readily separated using the same parameters as the 1.0 g load (Table 2, Run 4) on the 120 g Separation Column affording very similar F_2_G and FG yields. Increasing the sample load to 5 g using the same parameters and binary gradient as the 1 and 3 g loads, however, did not achieve F_2_G and FG separation on the 120 g separation column (Table 1, Run 6). The 5 g load separation was repeated with extending the 0–80% acetone in hexane gradient over 15 CV (Table 2, Run 7), an additional 5 CV relative to the 1–3 g sample load separations (Table 2, Run 3–6). Extending the gradient by 5 CV achieved F_2_G and FG in slightly lower yields than the previous runs. Also, this extended the total run time by ~20 min and increased the solvent used by ~1.2 L. The 7 g sample load separation using the 120 g Separation Column (Table 2, Run 8) successfully separated F_2_G and FG. However, the 10 g sample load (Table 2, Run 9) did not result in F_2_G and FG separation using identical conditions. It was theoretically possible to further slow the flow rate or further extend 0–80% acetone in hexane gradient to obtain 10 g sample load separation of F_2_G and FG using the 120 g RediSep Separation Column, although with the consequence of longer run times and large solvent volume use. Instead, separations of the higher sample loads (5, 7, and 10 g) were attempted using a larger (330 g) RediSep Separation Column.

Separations of 5, 7, and 10 g sample loads using a 24 g RediSep Load Column and 330 g RediSep Separation Column at a flow rate of 134 mL/min (~1/3 of the recommended flow rate) using a 0–80% acetone in hexane gradient over 15 CV (Table 2, Run 10–12). All three runs resulted in successful separation of F_2_G and FG, producing isolated yields similar to the 5 and 7 g sample loads separated with the 120 g Separation Column (Table 2, Run 7, 8). The exception was the 10 g load separation (Table 2, Run 12) that inexplicably resulted in lower F_2_G and FG yields, possibly due to post separation product mishandling. The 330 g Separation Column runs required even longer run times and much more solvent compared to the 120 g Separation Column runs.

After considering the Table 2 experimental results, which consumed ~54 g of the raw precipitate sample, Run 5 conditions were selected for purification of the remaining ~45 g of raw precipitate sample. Logistics of sample preparation (e.g., sample size, load column size, solute volume, time to aspirate load column to dryness), run time, time for solvent removal via rotary evaporation from the aliquots collected (300–500 mL), round bottomed flasks needed, and solvent waste generated were considered, and 3 g separations using Run 5 parameters allowed for convenient one flash chromatography run per day along with the subsequent solvent removal from the F_2_G and FG aliquots without undo solvent waste generation and storage for a single laboratory Hazardous Waste Satellite Accumulation Point. F_2_G and FG sample material from the unsuccessful separation runs were collected, dried, and purified in subsequent 3 g sample load separation runs. The 3 g sample load separation (Table 2, Run 5) was repeated 10 times resulting in an average yield of F_2_G 39.6 ± 1.7% and FG 10.4 ± 1.3% (50.0 ± 2.5% total) relative to the 3 g sample. The RediSep Load columns were aspirated to dryness after every use and were used up to seven times without noticeable deleterious effects. The RediSep Separation Columns that were stored in hexane in between separation runs could be used up to three times before the F_2_G and FG did not separate under a given run condition. When the RediSep Load and Separation Columns were aspirated to dryness and stored dry in between runs the columns could be reused up to seven times before the F_2_G and FG were observed to not separate under a given run condition. While the RediSep columns used herein are considered disposable by the manufacturer it was in the interest of cost that the columns were reused as often as possible.

Overall, combining the F_2_G and FG isolated from all flash chromatography runs, F_2_G and FG were isolated in a mass yields of 39.1 and 9.4 g, respectively (48.5 g total), from the 96.4 g of raw precipitate filtered from the feruloylated Enova Oil. Previously described separation methods (e.g., preparative HPLC, high-speed counter-current chromatography) to isolate F_2_G, FG, and similar phenylpropenoid glycerols as natural products from plant sources could only accommodate ~0.1–0.2 g of material [5] or the method had to be repeated over 40 times to purify 2–4 g of F_2_G or FG [26]. FG has been synthesized de novo and via the lipase catalyzed esterification of glycerol with ethyl ferulate or ferulic acid, but only in mg quantities identifiable by analytical HPLC [14,20] or purified by conventional column chromatography without yields reported [29]. The synthesis and isolation of F_2_G was previously reported in ~10 g yield as a precipitate collected from a similar lipase-catalyzed transesterification of ethyl ferulate with deacylated soybean oil; however, the purification was reported as a diethyl ether wash of the raw precipitate collected without FG being identified as a component [24]. Since both F_2_G and FG are sparingly soluble in diethyl ether, it is presumed that the FG was lost in the diethyl ether washes. Comparatively, the flash chromatography method reported herein can purify up to 2.0–3.0 g of F_2_G and FG per run in about an hour and was a necessary improvement over previously reported methods to be able to process ~95 g of raw material containing a mixture of F_2_G and FG in a timely and convenient manner.

### 4.3. Physicochemical Properties of F_2_G and FG

F_2_G and FG have been previously reported to be synthesized or isolated as colorless to yellow, oily liquids [14,29], and F_2_G has also been reported to have been isolated as a white solid [24]. F_2_G synthesized from the lipase-catalyzed transesterification of Enova Oil with ethyl ferulate and isolated by flash chromatography was a brittle, crystalline, white solid, and FG was isolated as a clear, light-yellow oil (Figure S4). On occasion, however, FG would form a chalky, white powder when dried from the hexane/acetone solutions (Figure S4). We controlled evaporation rates of FG saturated solutions in neat acetone and various binary acetone:hexane solutions (80:20–50:50) by slow, ambient temperature evaporation overnight and rapid evaporation under vacuum (11.6 Pa) with heating up to 60 °C to determine conditions for obtaining FG as a solid. Unfortunately, we were unable to definitively determine such optimal conditions for FG solid formation.

Theoretically, F_2_G and FG can both exist as two positional isomers. F_2_G can form the *sn*-1,3 positional isomer where the feruloyl moieties are covalently attached to the exterior, α-carbons of the glycerol molecule (See Figure S2 for carbon labels). The F_2_G *sn*-1(3),2 positional isomer consists of feruloyl moieties covalently attached to an exterior α- and the interior β-carbon of the glycerol molecule. The two F_2_G positional isomers result in distinctly different ^1^H NMR frequency splitting patterns. The F_2_G *sn*-1,3 isomer would be expected to exhibit a symmetric, α-, β-proton splitting pattern in a frequency ratio of 4:1 as found in Figure S2. More distinctive was the frequency of the β-proton of the unesterified β-carbon (4.1 ppm, Figure S2) vs. the expected frequency of the β-proton if the β-carbon was esterified with a feruloyl moiety (4.9–5.1 ppm) [30,31]. The absence of both the esterified β-carbon proton frequency peak and an α-, β-, γ-glycerol proton splitting pattern showed that the F_2_G isolated by flash chromatography was the *sn*-1,3 positional isomer. Similarly, FG can consist of the *sn*-1(3) and the *sn*-2 positional isomers where the feruloyl moiety is covalently attached to the α- or β-carbon, respectively. The FG *sn*-2 positional isomer would be predicted to exhibit a symmetrical α-, β-proton splitting pattern in a frequency ratio of 4:1 with the β-proton frequency being at 4.9–5.1 ppm as would be expected for an esterified β-carbon [32,33,34]. Instead, the FG isolated by flash chromatography exclusively exhibited a β-proton frequency signal at 3.9 ppm and a α-, β-, γ-glycerol proton splitting pattern in a 2:1:2 integration ratio (Figure S2). The FG isolated both as an oil and as a solid by flash chromatography was the *sn*-1(3) positional isomer. Acyl groups can migrate along the glycerol backbone, exchanging between the *sn*-1, -2, and -3 positions and is observed in mono- and diacylglycerols of which the kinetic rates of migration are dependent on time, temperature, presence of solvent, and acyl moiety [31,33,35,36,37,38]. The F_2_G and FG isolated by flash chromatography and stored at both room temperature and refrigerated exhibited no observable acyl migration as determined by ^1^H NMR over four weeks of storage, which was consistent with the lack of acyl migration found in *sn*-2 monoacylglycerols at ≤25 °C [34].

**Figure 4:**
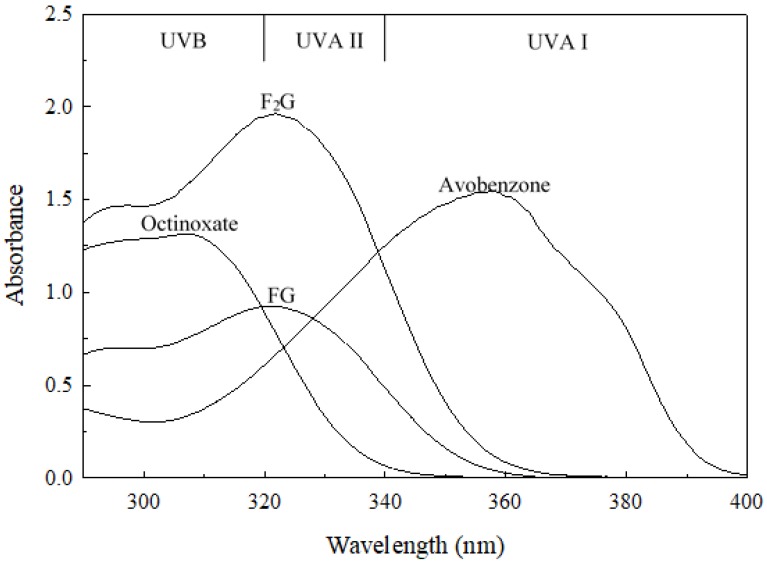
UV absorption spectra of F_2_G and FG in comparison to the commercial UV-absorbing ingredients Octinoxate and Avobenzone (50 μM solutions in acetonitrile). UVB, UVA II, and UVA I are defined by the U.S. Food and Drug Administration and the European Union Commission.

F_2_G and FG possess 4-hydrox-3-methoxycinnamoyl moieties (Figure 1) and these functional groups impart useful characteristics to the molecules. The aromatic hydroxyl group imparts antioxidant characteristics to the molecules, and the antioxidant capacity of F_2_G and FG has been well studied [29,39,40,41,42]. The conjugated electronic structure of the 3-methoxycinnamoyl functional group allows for electron delocalization across the aromatic ring, the desaturated aliphatic carbons, and the carbonyl group (Figure 1) with the methoxy group donating electron density to the aromatic ring, promoting the electron delocalization [43]. This allows the molecule to absorb ultraviolet (UV) radiation and dissipate the energy as heat. F_2_G and FG are structurally similar to methoxycinnamates used as commercial UV-absorbing compounds in retail formulations (Figure S5) [43]. Figure 4 shows the UV absorption spectra of F_2_G and FG compared to the commercial UVB-absorbing ingredient, Octinoxate, and the commercial UVA-absorbing ingredient, Avobenzone. For the purpose of characterizing retail products claiming Sun Protection Factors and Broad Spectrum Protection the U.S. Food and Drug Administration divides the UV spectrum into UVC (<289 nm), UVB (290–320 nm), UVA II (321–340 nm), and UVA I (341–400 nm) [44]. F_2_G and FG possessed a λ_max_ 321 in acetonitrile solutions (Figure 4) and a λ_max_ 328 in ethanol (data not shown), their strongest absorbances were in the UVA II region and extended into the UVB region. F_2_G and FG did not strongly absorb in the UVA I region. The UV absorbance spectra and λ_max_ of F_2_G and FG were very similar to that of feruloylated soybean oil reported in ethanol and acetonitrile solutions [45]. The extinction coefficient, ε, of F_2_G (10–50 µM) and FG (10–100 µM) were determined in ethanol (Figure S6) and acetonitrile solutions (Figure S7) as the slope of the linear regression of absorbances at λ_max_, *n* = 3 trials. FG had an ε 19.200 ± 154 M^−1^ cm^−1^ and 18,500 ± 46 M^−1^ cm^−1^ in ethanol and acetonitrile solutions, respectively. F_2_G had an ε 40,000 ± 60 M^−1^ cm^−1^ and 37,200 ± 125 M^−1^ cm^−1^ in ethanol and acetonitrile solutions, respectively, nearly double the extinction coefficients of FG. This was expected since F_2_G possessed twice the molar equivalents of the feruloyl UV chromophore than FG. F_2_G and FG possessed extinction coefficients higher than those reported for feruloylated soybean oil, 11,200 ± 58 and 13,400 ± 69 M^−1^ cm^−1^, in ethanol and acetonitrile solutions, respectively [45]. Although FG did not absorb as strongly as the commercial UV absorbers, the F_2_G extinction coefficients were much higher than both Octinoxate, 24,000–25,400 M^−1^ cm^−1^, reported in ethanol and acetonitrile solutions and Avobenzone, 34,000–30,800 M^−1^ cm^−1^, reported in ethanol and acetonitrile solutions [45,46,47]. Retail formulations often contain multiple UV-absorbing ingredients, such as Octinoxate and Avobenzone, to protect against exposure to the full 240–400 nm UV absorbance spectrum. The UV-absorbing characteristics of F_2_G and FG suggest that they could function as fungible replacements for commercial UVB-absorbing ingredients or be used in conjunction with commercial UV absorbers to enhance the UVA II absorbance of formulations.

**Figure 5:**
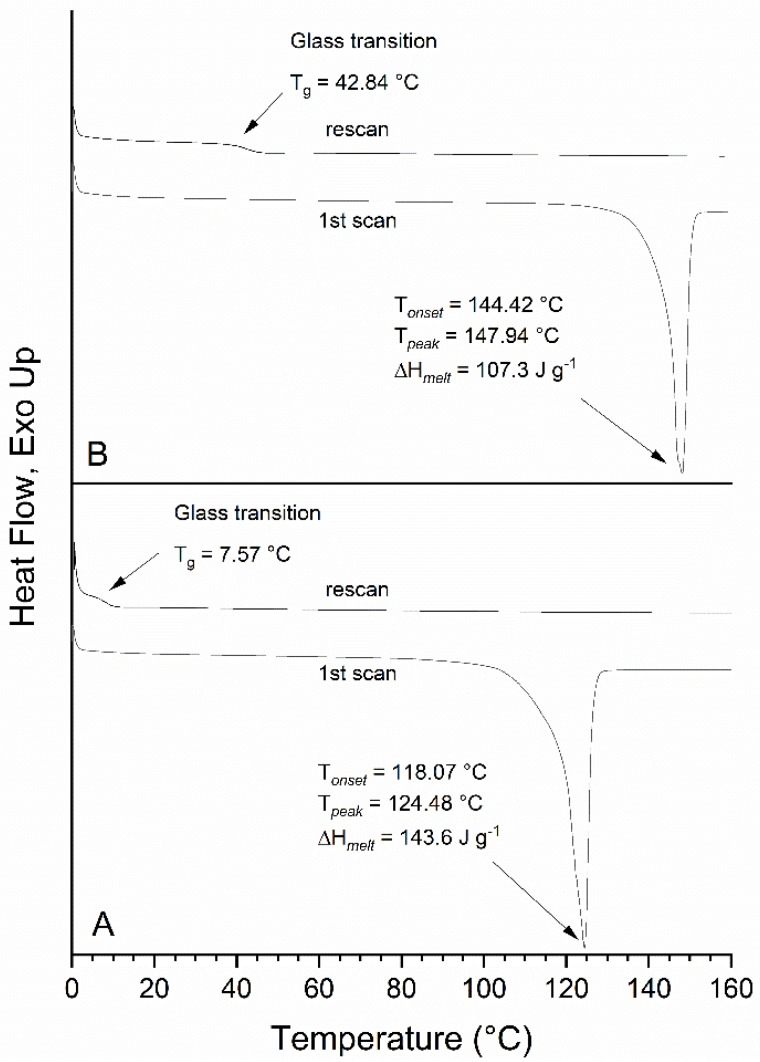
Differential scanning colorimetry of pure: (**A**) FG and (**B**) F_2_G.

The thermal behavior of FG and F_2_G was determined by DSC and their thermograms are shown in Figure 5. FG showed a single endothermic melting transition with an onset temperature of T*_onset_* = 118.07 °C and melting peak at T*_peak_* = 124.48 °C (ΔH*_melt_* = 143.6 J g^−1^). As expected, the additional ferulate group increased the melting point of the material relative to FG as shown by the F_2_G thermogram that had an onset temperature of T*_onset_* = 144.42 °C and an endothermic melting transition at T*_peak_* = 147.94 °C (ΔH*_melt_* = 107.3 J g^−1^). Interestingly, the cooling cycle showed no endothermic transitions indicating recrystallization of the materials and the subsequent rescan of both FG and F_2_G only showed a small endothermic event at 7.8 °C and 42.8 °C, respectively, suggesting a glass transition of an amorphous material. From the rescan it is clear the crystallinity of both these compounds was lost due to the thermal processing of the samples. To further examine these samples, the FG and F_2_G DSC samples, having been exposed to 160 °C, were examined by ^1^H NMR and the spectra (not shown) demonstrated that acyl migration had occurred in both FG and F_2_G to form the *sn*-2 and *sn*-1,2 positional isomers, respectively. The presence of acyl migration products within FG and F_2_G likely precluded crystallization of the compounds.

## 5. Conclusions

A flash chromatography method using a binary gradient of hexane and acetone was optimized for the separation of the natural compounds F_2_G and FG that were obtained as a byproduct mixture from the lipase-catalyzed transesterification of ethyl ferulate and commercial vegetable oil, Enova Oil (≥80% DAG). Over 95.0 g of the crude byproduct mixture was processed by flash chromatography to yield 39.1 g of F_2_G and 9.4 g of FG. This method was an improvement from the preparative HPLC and column chromatography methods reported to purify milligram quantities of F_2_G and FG mixtures or used to individually isolate small gram quantities of F_2_G or FG to date. F_2_G and FG were isolated as the *sn*-1,3 and *sn*-1 positional isomers, respectively, and did not exhibit acyl migration during the flash chromatography purifications or during storage. F_2_G and FG possessed UV-absorbing characteristics, λ_max_ 328 nm and ε 40,000 ± 60 and 19,200 ± 154 M^−1^ cm^−1^, respectively, in ethanol solutions, making them suitable as fungible replacements for commercial UVB absorbers or UVA II-absorbing enhancers when used with commercial UVB- and UVA-absorbing compounds.

## Figures and Tables

**Table 1:** Manufacturer’s (Teledyne-Isco) specifications and recommended parameters for the RediSep Rf Gold (High Performance) Silica Gel (20–40 microns) Disposable Flash Chromatography Columns.

Size	Column Volume	Flow Rate	Default Slope-Based Peak Width Detection	Max. Pressure	Sample Loading ^a^
(g)	(mL)	(mL/min)	(min)	(psi | kPa)	(g)
4	4.8	18	0.5	600 | 4137	0.020–0.40
12	16.8	30	1	400 | 2758	0.060–1.2
24	35.9	35	1	350 | 2413	0.12–2.4
120	192.0	85	4	225 | 1551	0.60–12.0
220	243.0	150	4	150 | 1034	1.10–22.0
330	443.0	200	4	150 | 1034	1.65–33.0

^a^ Recommended minimum and maximum mass of sample to use for optimal separation.

**Table 2:** Flash chromatography parameters and separation results for the purification of F_2_G and FG.

	Column Size ^a^	Sample Load ^b^	Product Yield ^c^			Run Parameters				
Run	Load	Separation	Solute	Solvent	F_2_G	FG	Total	Flow Rate	Length ^d^	Solvent
(#)	(g)	(g)	(g)	(mL)	(g | %)	(g | %)	(%)	(mL/min)	(CV | min)	(L)
1	4	24	0.100	1.0	-	-	-	35	16 | 33 ^g^	1.2
2 ^e^	4	24	0.100	1.0	0.038 ± 0.0025 | 38 ± 2.5	0.0094 ± 0.0002 | 9.4 ± 0.2	47.4 ± 2.4	20	16 | 33 ^g^	0.7
3	12	120	1.000	5.0	0.383 | 38.3	0.088 | 8.8	47.1	85	10 | 41 ^h^	3.5
4	12	120	1.000	5.0	0.400 | 40.0	0.090 | 9.0	49.0	57	10 | 52 ^h^	3.0
5 ^f^	12	120	3.000	10.0	1.188 ± 0.052 | 39.6 ± 1.7	0.313 ± 0.038 | 10.4 ± 1.3	50.0 ± 2.5	57	10 | 52 ^h^	3.0
6	12	120	5.000	10.0	-	-	-	57	10 | 52 ^h^	3.0
7	12	120	5.000	15.0	1.730 | 34.6	0.433 | 8.7	43.3	57	15 | 74 ^i^	4.2
8	24	120	7.000	15.0	2.639 | 37.7	0.627 | 9.0	46.7	57	15 | 74 ^i^	4.2
9	24	120	10.00	22.0	-	-	-	57	15 | 74 ^i^	4.2
10	24	330	5.000	15.0	1.760 | 35.2	0.414 | 8.3	43.5	134	15 | 100 ^i^	13.4
11	24	330	7.000	17.0	2.615 | 37.4	0.602 | 8.6	46.0	134	15 | 100 ^i^	13.4
12	24	330	10.00	22.0	2.457 | 24.6	0.727 | 7.3	31.9	134	15 | 100 ^i^	13.4

^a^ RediSep Rf Gold (High Performance) Silica Gel (20–40 microns) Disposable Flash Chromatography Columns (see Table 1). ^b^ Solute = mass of the precipitate collected from the transesterification of ethyl ferulate and Enova Oil used for the run. Solvent = acetone used to dissolve the solute for loading onto the Load Column. ^c^ Percent yields of F_2_G and FG are relative to the mass of solute. Dash = product did not base line separate during run. ^d^ CV = column volumes. Run length (min) includes the column equilibration time in addition to the separation run time. ^e^ *n* = 3 runs with product yield reported as the mean ± one standard deviation from the mean. ^f^ *n* = 10 runs with product yield reported as the mean ± one standard deviation from the mean. ^g^ Solvent gradient (A = hexane and B = acetone): 100% A for 3 CV (column equilibration), 0–80% B over 13 CV, 80% B for 2 CV, and 100% A for 1 CV. ^h^ Solvent gradient (A = hexane and B = acetone): 100% A for 3 CV (column equilibration), 0–80% B over 7 CV, 80% B for 1 CV, and 100% A for 2 CV. ^i^ Solvent gradient (A = hexane and B = acetone): 100% A for 3 CV (column equilibration), 0–80% B over 12 CV, 80% B for 1 CV, and 100% A for 2 CV.

## Supplementary Materials

The following are available online at https://www.mdpi.com/2409-9279/3/1/8/s1. Table S1: CombiFlash Rf200i flash chromatography of ethyl ferulate standard at varying flow rates, 5–27 mL/min, Figure S1: Photograph of the CombiFlash Rf 200i flash chromatography system, Figure S2: ^1^H NMR (500 MHz, *d6-actone) of FG and F_2_G, Figure S3: CombiFlash Rf200i flash chromatography of ethyl ferulate standard at varying flow rates, 5–27 mL/min, Figure S4: Photograph of F_2_G and FG purified by flash chromatography, Figure S5: Chemical structures of the commercial UV-absorbing ingredients, Figure S6: Absorbance extinction coefficient (ε, M^−1^ cm^−1^) of FG and F_2_G in ethanol, and Figure S7: Absorbance extinction coefficient (ε, M^−1^ cm^−1^) of FG and F_2_G in acetonitrile.
