# Supplementary material for: Enzymatic Synthesis and Flash Chromatography Separation of 1,3-Diferuloyl-sn-Glycerol and 1-Feruloyl-sn-Glycerol
Source: Methods Protoc. 2020 Jan 16;3(1):8. doi: 10.3390/mps3010008 (PMC7189784; doi:10.3390/mps3010008)
Supplement: Supplementary file 1 [file mps-03-00008-s001.pdf]

Supplemental Material

Enzymatic Synthesis and Flash Chromatography Separation of the Natural Phenylpropenoids, 1,3-Diferuloyl-*sn*-Glycerol and 1-Feruloyl-*sn*-Glycerol

David L. Compton [1,*], Michael Appell [2], James A. Kenar [3] and Kervin O. Evans [1]

[1] Renewable product Technology Research Unit, United States Department of Agriculture[†], Agricultural Research Service, National Center for Agricultural Utilization Research, 1815 N. University Street, Peoria, IL 61604, USA; david.compton@usda.gov; kervin.evans@usda.gov

[2] Mycotoxin Prevention and Applied Microbiology, United States Department of Agriculture, Agricultural Research Service, National Center for Agricultural Utilization Research, 1815 N. University Street, Peoria, IL 61604, USA; michael.appell@usda.gov

[3] Functional Foods Research Unit, Unaited States Department of Agriculture, Agricultural Research Service, National Center for Agricultural Utilization Research, 1815 N. University St., Peoria, IL, 61604, USA; jim.kenar@usda.gov

[*] Correspondence: David.Compton@usda.gov; Tel.: +1 (309) 681-6321

[†] Mention of trade names or commercial products in this publication is solely for the purpose of providing specific information and does not imply recommendation or endorsement by the United States Department of Agriculture (USDA). USDA is an equal opportunity provider and employer.

Table S1. CombiFlash Rf200i flash chromatography of ethyl ferulate standard at varying flow rates, 5 – 27 mL/min. See Figure S3 for experimental parameters.

	Peak Base Width		ELSD Peak		
Flow Rate	UV	ELSD	Off Set[b]	Run Time	
(mL/min)	(CV[a])	(CV)	(CV)	(CV)	(min.)
5.0	0.75	0.90	+1.00	20.0	19.2
10.0	0.88	0.98	+0.71	20.0	9.6
18.0	1.42	1.78	−0.15	20.0	5.3
27.0	1.78	2.58	−0.31	20.0	3.6

[a] The CombiFlash Rf200i measures run time in Column Volumes (CV) and minutes. The CV of the RediSep RF Gold 4-g Silica Gel Disposable column was 1 CV = 4.8 ml at 18 ml/min default flow rate.
[b] The difference between the start of the ELSD peak detection before (+) or after (-) the start of the UV peak detection.

Load Column

Separation Column

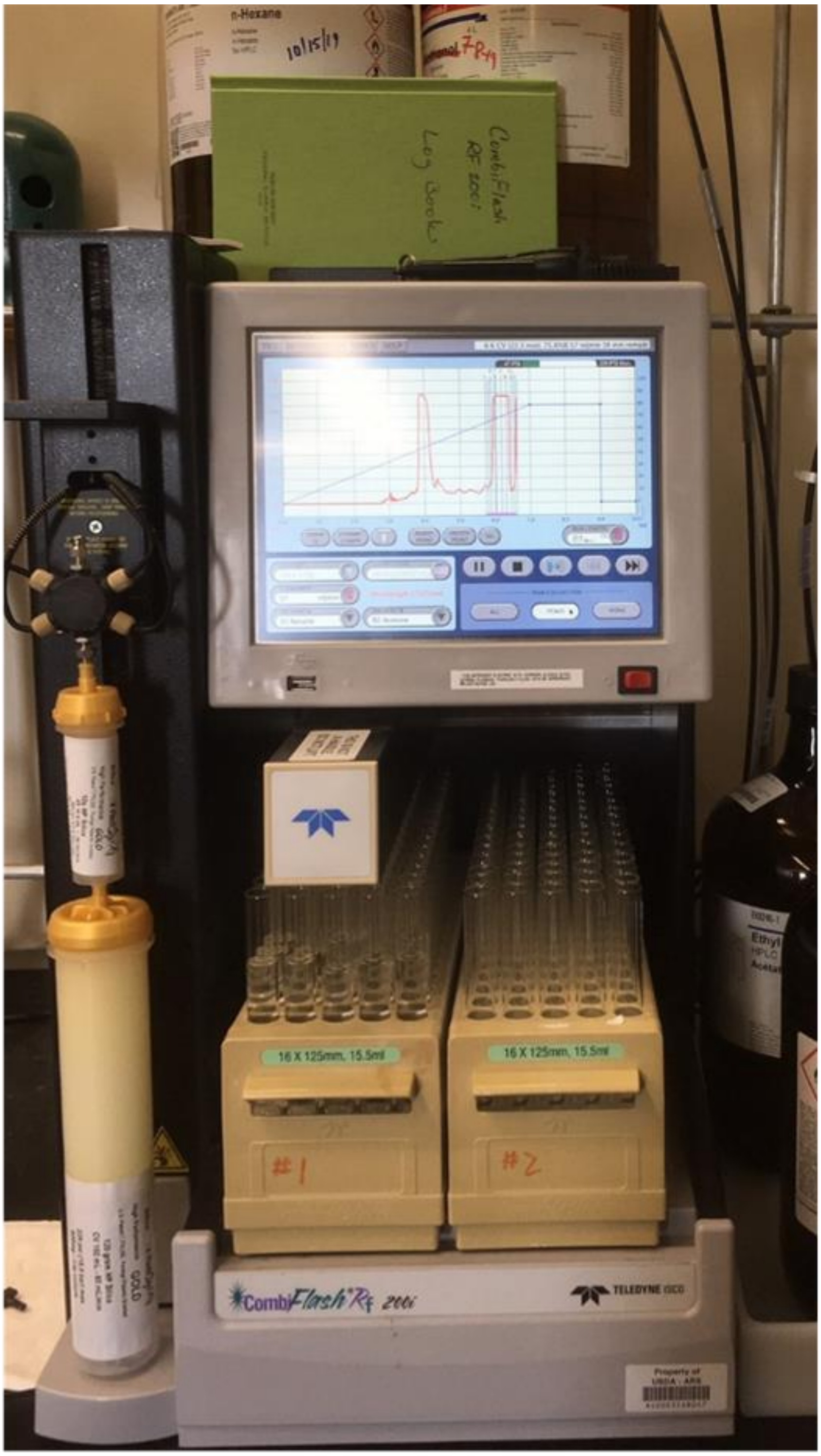

Figure S1. Photograph of the CombiFlash R_f 200i flash chromatography system with a 4-g RediSep RF Gold Silica Gel Disposable Flash Chromatography Column (20 – 40 microns) as the "load column" containing the raw precipitate plumbed above a 24-g RediSep RF Gold Silica Gel Disposable Flash Chromatography Column (20 – 40 microns) "separation column.".

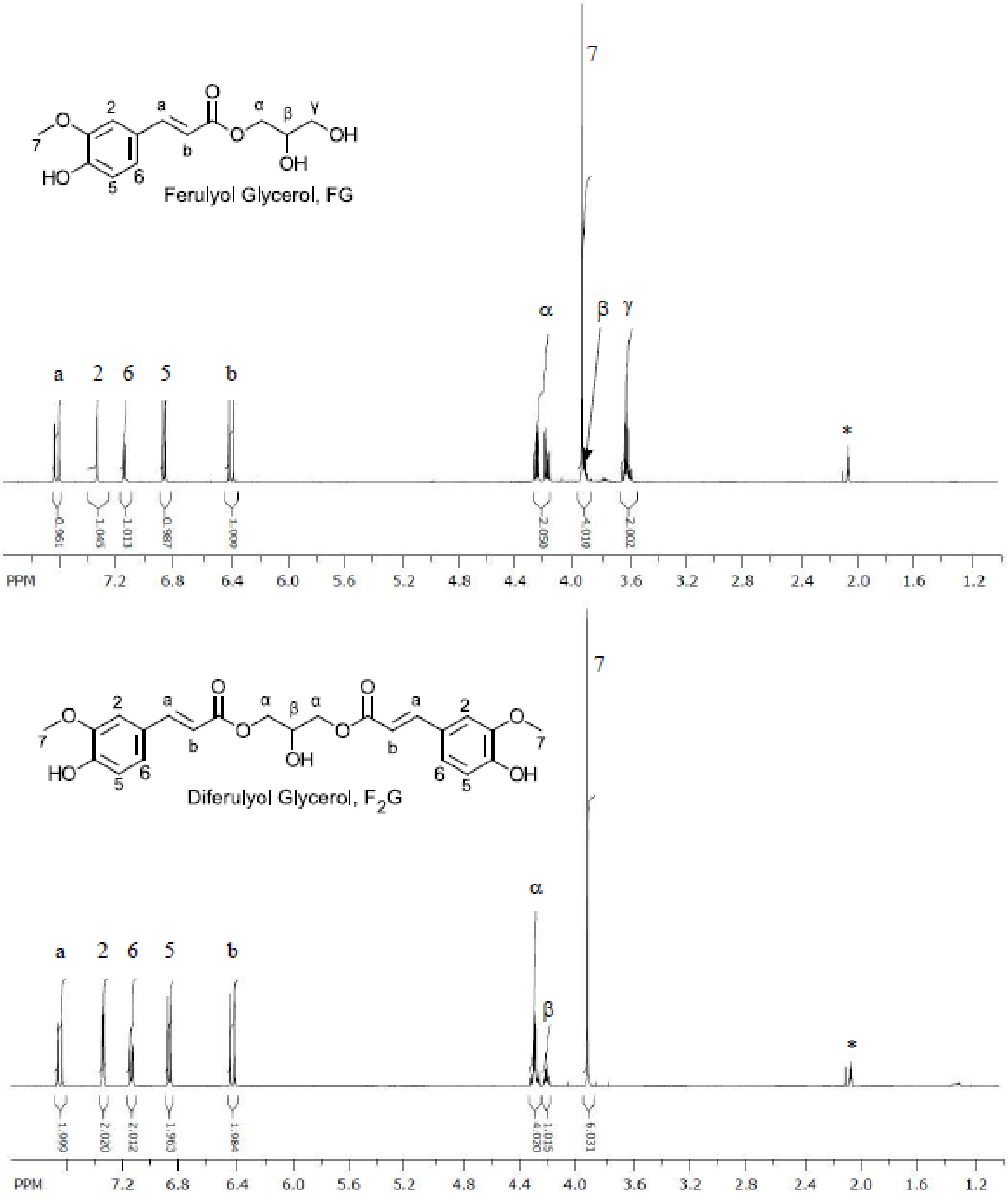

Figure S2. ^{1}H NMR (500 MHz, *d$_6$-actone) of FG and F$_2$G.

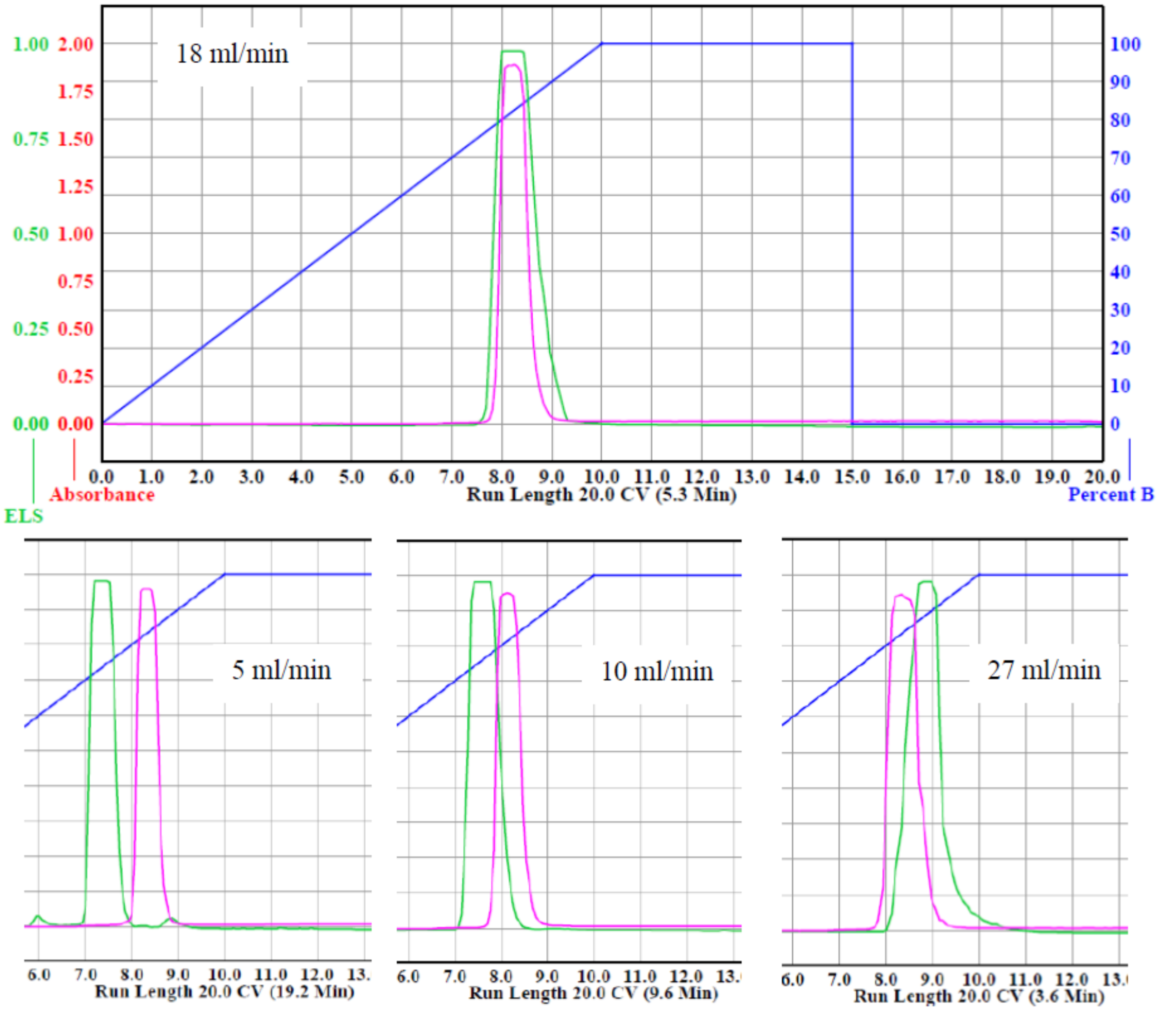

was monitored by UV (325 nm, *left-red axis, purple trace*) and ELSD (left-*green axis, green trace*).

Figure S3. CombiFlash Rf200i flash chromatography of ethyl ferulate standard at varying flow rates, 5 – 27 ml/min. Ethyl ferulate standard, 10 mg, dissolved in 250 l acetone was 1-ml disposable syringe injected onto a RediSep RF Gold 4-g Silica Gel Disposable column (1 Column Volume, CV, = 4.8 ml at 18 ml/min default flow rate) and aspirated to dryness under vacuum for 30 min. The column was developed as follows: liquid injection mode of 3.0 CV equilibration with 100 % (v:v) hexane (solvent A), followed by 0 – 100 % gradient (*blue line*) with acetone (solvent B, *right-blue axis*) for 10 CV, followed by 5 CV of 100 % acetone, followed by 5 CV of 100 % hexane. The signal.

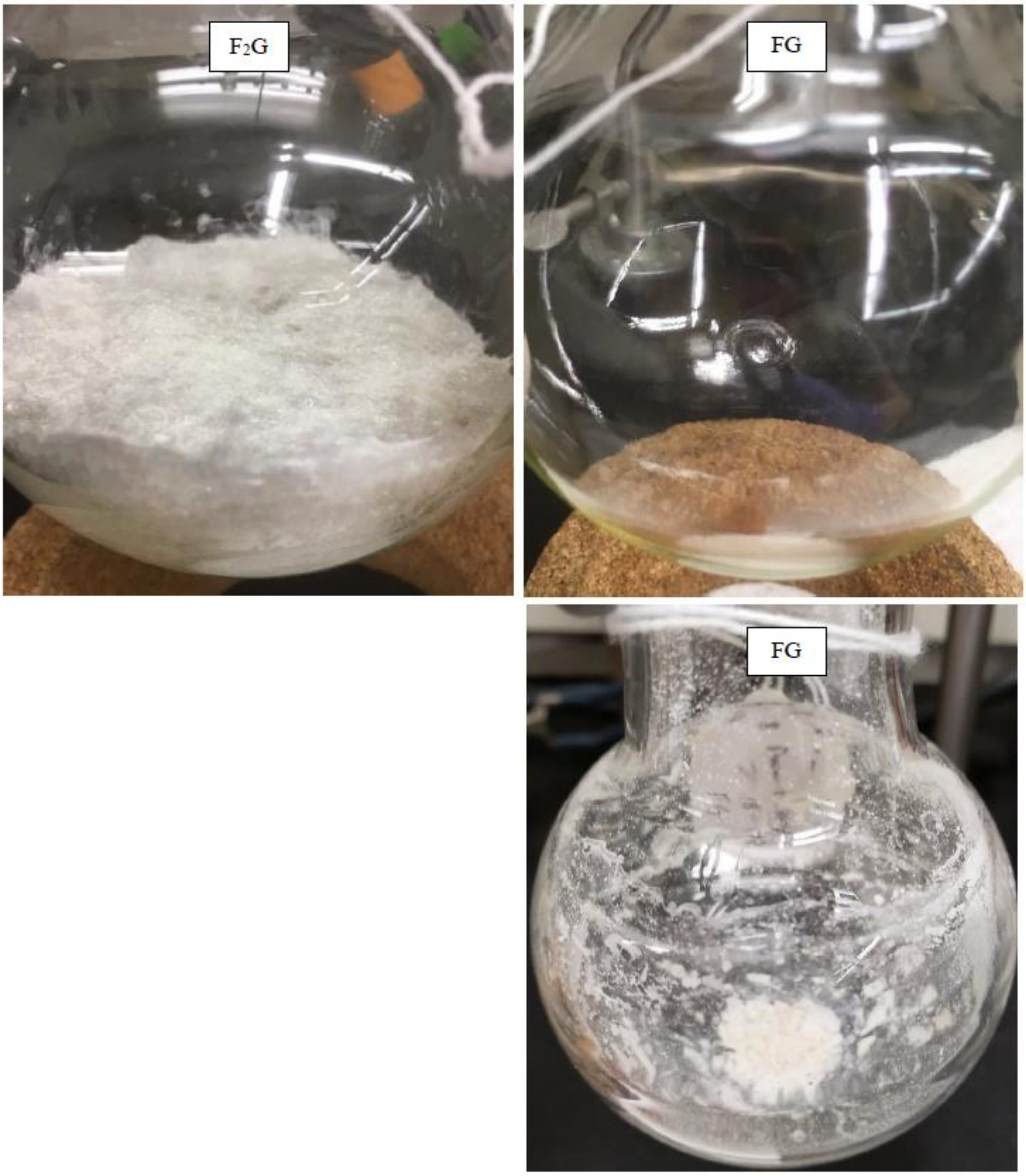

Figure S4. Photograph of F_2G and FG purified by flash chromagraphy of the crude precipitate (3 g) collected from the lipase-catalyzed transesterification of Enova Oil with ethyl ferulate.

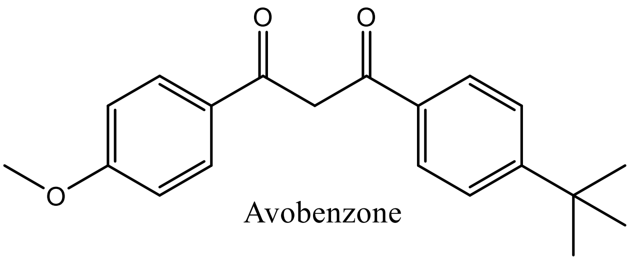

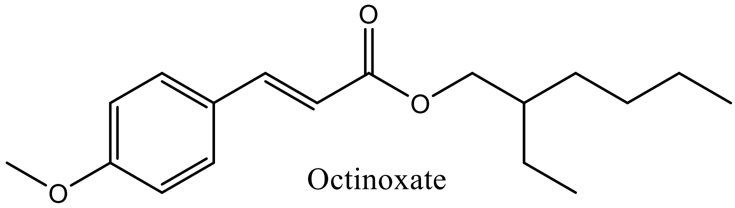

Figure S5. Chemical structures of the commercial UVB absorbing ingredient, Octinoxate, and the commercial UVA absorbing ingredient, Avobenzone.

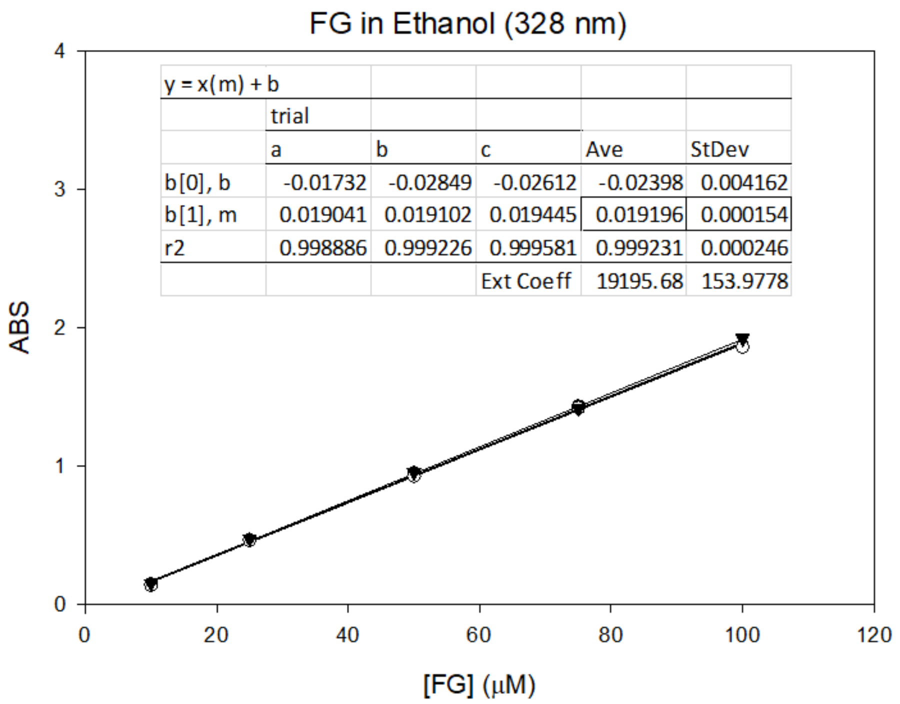

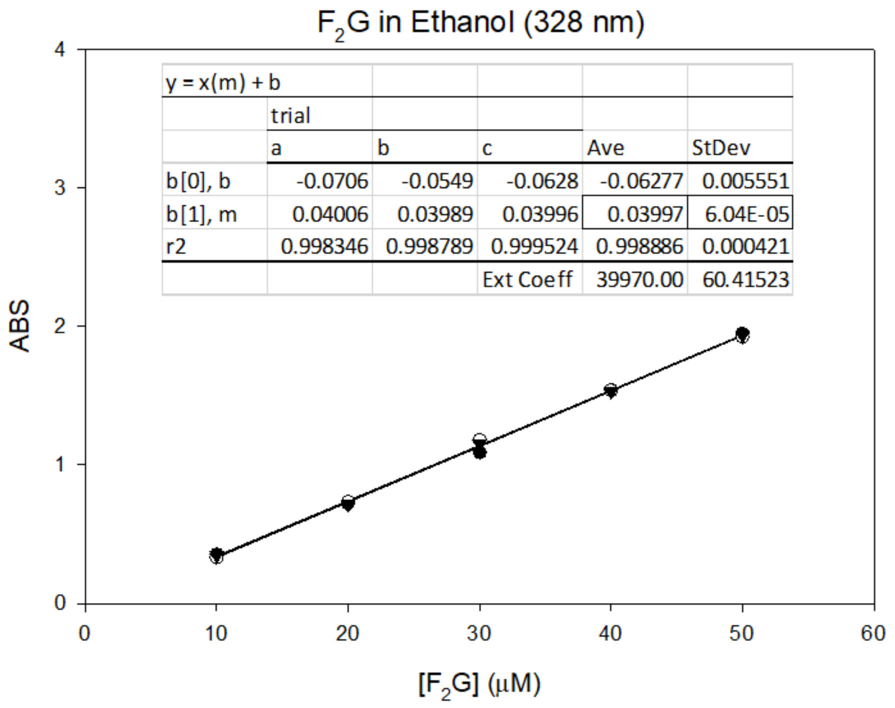

Figure S6. Absorbance extinction coefficient (ε, M^{-1} cm^{-1}) of FG and F_2G in ethanol solutions determined as the slope of the linear regression of absorbance at λ_{max}, n = 3 trials.

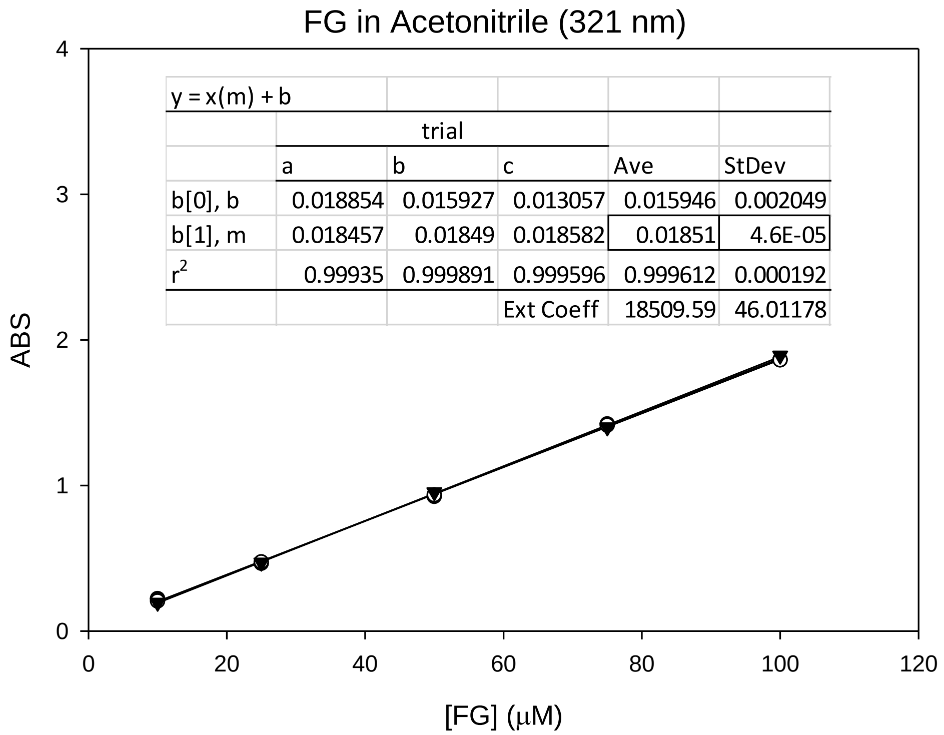

y = x(m) + b					
	trial				
	a	b	c	Ave	StDev
b[0], b	0.018854	0.015927	0.013057	0.015946	0.002049
b[1], m	0.018457	0.01849	0.018582	0.01851	4.6E-05
r^2	0.99935	0.999891	0.999596	0.999612	0.000192
			Ext Coeff	18509.59	46.01178

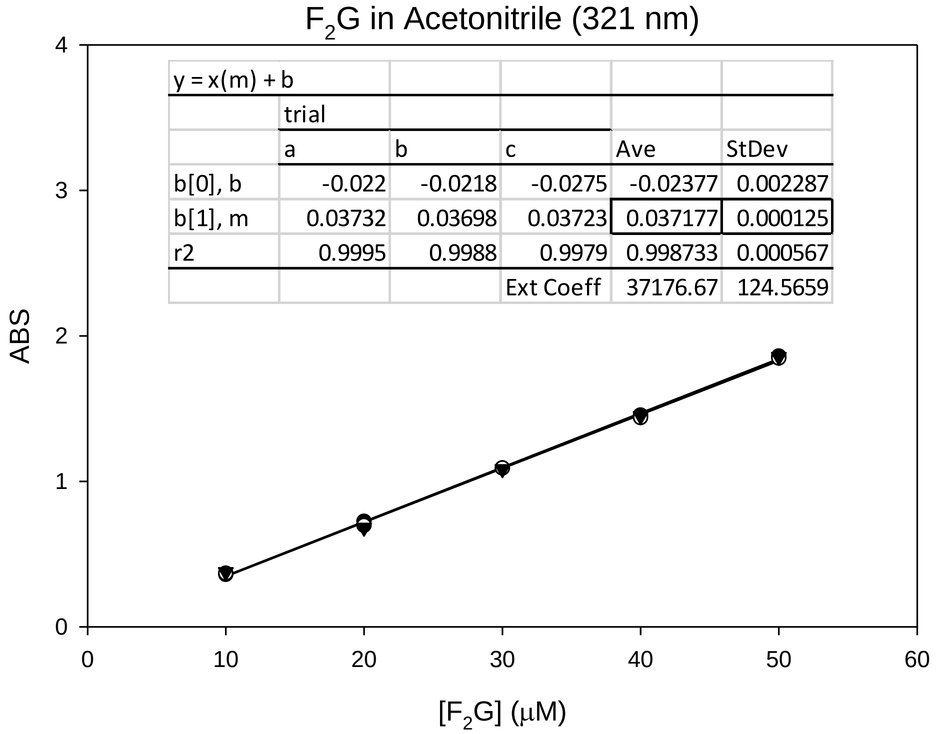

y = x(m) + b					
	trial				
	a	b	c	Ave	StDev
b[0], b	-0.022	-0.0218	-0.0275	-0.02377	0.002287
b[1], m	0.03732	0.03698	0.03723	0.037177	0.000125
r2	0.9995	0.9988	0.9979	0.998733	0.000567
			Ext Coeff	37176.67	124.5659

Figure S7. Absorbance extinction coefficient (ε, M^{-1} cm^{-1}) of FG and F_2G in acetonitrile solutions determined as the slope of the linear regression of absorbance at λ_{max}, n = 3 trials.
